# Antioxidant System Activity in Roots and Shoots of Bean Cultivars in Response to Seed Treatment with Auxin as a Potential Model of Interaction with Endophytic Bacteria

**DOI:** 10.3390/plants13233365

**Published:** 2024-11-29

**Authors:** Svetlana Garipova, Viktoriia Matyunina, Aelita Chistoedova, Oksana Markova, Alsu Lubyanova, Oksana Lastochkina, Ekaterina Pedash, Azamat Avalbaev, Lyudmila Pusenkova

**Affiliations:** 1 Institute of Nature and Human, Ufa University of Science and Technology, Zaki Validy Str. 32, 450076 Ufa, Russia; viktoria030700@mail.ru (V.M.); achistoyedova@mail.ru (A.C.); o-ksana@list.ru (O.M.); pedashkatia@mail.ru (E.P.); 2 Institute of Biochemistry and Genetics, Subdivision of the Ufa Federal Research Center of the Russian Academy of Sciences, Pr. Oktyabrya 71, 450054 Ufa, Russia; lubyanova555@mail.ru (A.L.); oksana.lastochkina@ufaras.ru (O.L.); avalbaev@yahoo.com (A.A.); 3 Bashkir Research Institute of Agriculture, Subdivision of the Ufa Federal Research Center of the Russian Academy of Sciences, R. Zorge Str. 19, 450059 Ufa, Russia; l.pusenkova@mail.ru

**Keywords:** *Phaseolus vulgaris* L., seedlings, growth, exogenous IAA, endophytic bacteria, hydrogen peroxide, catalase, malondialdehyde, plant–microbe interaction

## Abstract

Plant growth-promoting endophytic bacteria (PGPEB), producing auxins, are offered for a promising eco-friendly crop production. Precise bacterial strain selection is essential to ensure consistent and effective plant growth and resilience. Creating a model for the optimal dose-dependent interactions between PGPEB and hosts is necessary for understanding the mechanisms of high-precision selection of the inoculant composition to enhance bacterial preparations’ efficacy. This study investigated the impact of pre-sowing treatment with exogenous auxin indole-3-acetic acid (IAA) at various concentrations (0, 10, 1, 0.1, 0.01, 0.001, 0.0001, 0.00001 mg L^−1^) on the growth and antioxidant responses of three cultivars (*cvs*) of *Phaseolus vulgaris* L. (bean): Ufimskaya, Elsa, and Zolotistaya. The findings showed dose-dependent and cultivar-specific responses of 7-day-old bean seedlings to exogenous IAA. Ufimskaya *cv* exhibited significant increases in shoot, main root, and total root lengths at 0.001 mg L^−1^ IAA, while higher and lower concentrations inhibited growth. The reduced catalase (CAT) activity in roots and the elevated CAT activity in shoots correlated with shoot length and total root length of Ufimskaya *cv*. Importantly, the growth parameters exhibited weak or no correlations with malondialdehyde (MDA) and H_2_O_2_ content in roots and shoots, which is a peculiarity of the Ufimskaya *cv* response to exogenic IAA in contrast to the shown earlier response to inoculation with endophytes. The growth of only the main root of Elsa *cv* peaked at 0.1 mg L^−1^ IAA, and there were neutral or inhibitory effects with other concentrations. The positive correlation between CAT activity in shoots and the main root length and total root length as well as positive correlation between MDA content in roots and the total root length of Elsa cultivar were revealed. The shoot length and total root length of Zolotistaya *cv* were neutral or negatively responded to all concentration IAA, but the number of roots increased by 2–4 times. For Zolotistaya *cv*, positive correlations were observed between CAT activity in roots and the length of the main root and the total root length. Overall, these cultivar-specific antioxidant responses to exogenous IAA may help create models for optimal dose-dependent interactions between auxin-producing PGPEB and plants, enhancing the effectiveness of microbial preparations for consistent bean growth promotion.

## 1. Introduction

Beans (*Phaseolus vulgaris* L.) are a valuable high-protein food crop, containing a high proportion of vitamins and microelements [1,2]. Bean cultivars adapted to local environmental conditions and resistant to adverse abiotic factors are being developed and tested around the world [3,4]. Metabolic and physiological parameters, as well as genetic markers, associated with bean yield, are being sought [5,6]. In the conditions of the Russian Federation, significant adaptive factors are the speed of ripening during a short growing season, resistance to pests and diseases, drought, low temperatures, etc. [7,8]. The adaptive potential of existing cultivars can be significantly increased by interaction with beneficial endophytic microorganisms, asymptomatically inhabiting the internal tissues of plants, which are known to enhance the growth of plants directly and indirectly [9,10,11]. A detailed molecular and biochemical understanding of endophyte entry and colonization strategy in the host would better help in manipulating crop productivity under changing climatic conditions [12].

One of the factors of growth-stimulating activity of beneficial bacteria associated with plants is their production of phytohormones, particularly indoleacetic acid (IAA), which is considered the major mechanism by which these bacteria facilitate plant growth [13]. It has been shown that the production of bacterial IAA induces root growth, promotes an increase in the survival rate of inoculated plantlets, and affects root architecture, nutrient uptake, and resistance to various abiotic stresses such as drought, salinity, and heavy metal toxicity [14,15]. At the same time, the inoculation of plants with auxin-producing bacteria may lead to a wide variety of responses, ranging from effective growth promotion to growth restriction, depending on the sensitivity of different plants to auxin levels [16]. Not only plant species, but also cultivars, can differ in their susceptibility to exogenous auxin concentrations [17]. Supplementing plants with additional auxin synthesized by bacteria, could potentially alter the endogenous phytohormone level to either optimal or not optimal and lead to significant physiological changes in plants associated with these bacterial strains [18,19]. Seed treatment of peas with auxin-producing endophytic bacteria at cell doses exceeding the optimum delayed plant growth reduced seed productivity [20]. Thus, the concentration of auxin is an important factor that influences a crop’s response to bacterial biofertilizers. This must be considered for the practical use of such bacterial biofertilizers, in order to achieve better and more consistent results from inoculation [16].

Auxins are known to play a role not only as growth regulators in plant development but also in plant–microbe interactions [21,22]. Elevated auxin levels or enhanced auxin signaling can promote disease development in some plant–pathogen interactions [23]; however, the appropriate dose of auxin is also necessary for forming effective symbiotic relationships [19]. Since the optimal concentrations of IAA are plant species- and cultivar-specific, hence determining the concentrations of exogenous IAA, as a model of IAA-producing bacteria interactions with plants, can help create the formulations for biopreparations.

It was shown that there is a feedback effect between the plant immune system and bacterial auxin. When colonizing a plant, bacteria trigger the immune response of the plant roots by initiating the production of reactive oxygen species (ROS). This response, in turn, stimulates the bacteria to synthesize auxin, which further promotes plant growth [24,25]. An important factor in perception and signaling in plant defense is the production and regulation of ROS, which might be controlled by some bacteria by producing antioxidant enzymes, such as catalases (CAT), superoxide dismutase (SOD), and glutathione-S-transferases, among others [26]. The effectiveness of ROS detoxification processes during plant–microbe interactions can be assessed by measuring the level of hydrogen peroxide (H_2_O_2_), malondialdehyde (MDA) in cells, as well as the activity of enzymes such as CAT and peroxidase (POD) [27]. In this regard, the response of the plant antioxidant system to seed treatment with different concentrations of exogenous auxin is of interest as a potential model for understanding the influence of auxin-producing bacteria on seedling growth parameters.

Previously, in a model system, it was found that a decrease in MDA levels in the tissues of inoculated 7-day-old bean sprouts, compared to the control, can serve as a biochemical marker for effective cultivar-strain combinations [28]. Using the Ufimskaya cultivar (*cv*), which specifically responded to inoculation with different doses of endophytic bacteria *B. subtilis* 26D and 10-4, it was shown that, alongside a reduction in MDA content in the roots, a high concentration of H_2_O_2_ in the roots and shoots of 7-day-old seedlings closely correlated with increased seed productivity of inoculated plants under field conditions [29]. To verify the predictors of effective symbiosis identified using the Ufimskaya *cv*, it is necessary to test them in the interactions involving several strains and different bean cultivars. The Ufimskaya, Zolotistaya, and Elza *cvs* were characterized by differences in their adaptive properties [8]. Following the inoculation with auxin-producing endophytic bacteria *B. subtilis* 26D and 10-4, the Ufimskaya and Zolotistaya *cvs* exhibited different phenotypic responses both under normal and stress conditions in model experiments [28,30] and during inoculation in the field [31,32]. Understanding the adaptive properties of the cultivars and the features of their interaction with model strains of auxin-producing bacteria, would be important to evaluate the response of the cultivars’ antioxidant systems to seed treatment with a gradient of exogenous auxin concentrations in a system without bacteria. This would be helpful in order to reveal subsequently, whether the contribution of auxin-producing bacteria to the formation of endophytic symbiosis is associated with the direct growth-stimulating action of bacterial phytohormones (i.e., auxin) or with other effects of these relationships.

Thus, this study aimed to evaluate the effects of varying concentrations of exogenous IAA on the growth and antioxidant system responses of different bean genotypes (Ufimskaya, Zolotistaya, and Elza *cvs*) in the early stages of ontogenesis. Based on these data, a correlation analysis between the growth and biochemical indices of plants and a comparison of different cultivars will be conducted.

## 2. Results

### 2.1. The Effect of Treating Ufimskaya Cultivar Seeds with a Gradient of Exogenous Auxin Concentrations on Growth Parameters, Antioxidant System Indicators, and the Correlation Relationships Between Them

**Figure 1:**
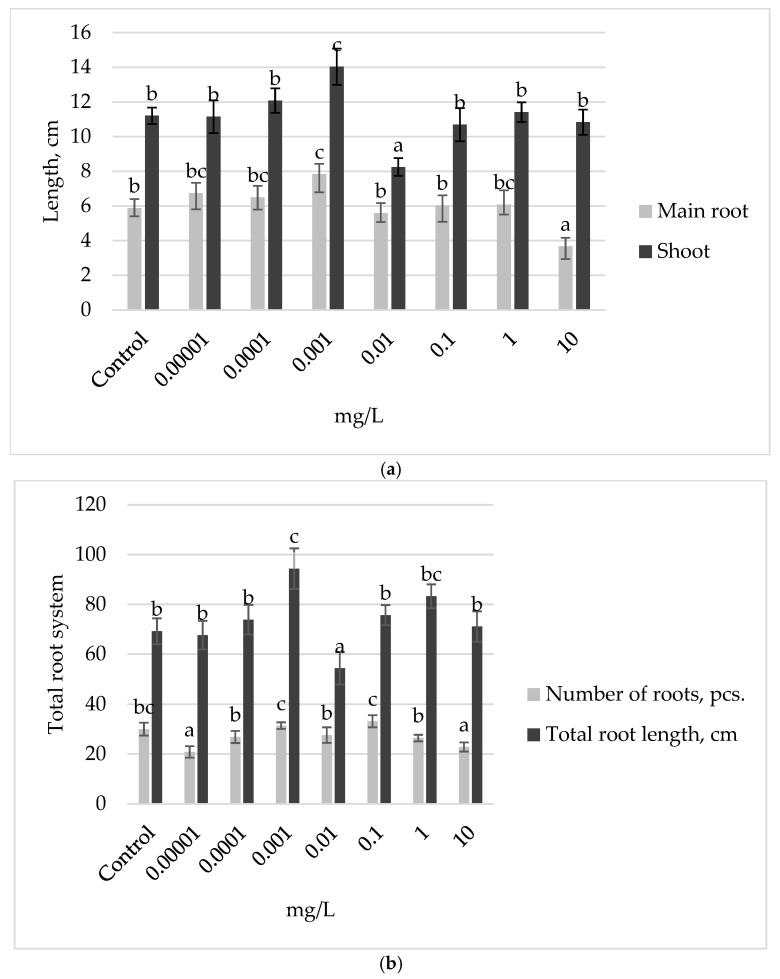
Growth parameters of 7-day-old bean seedlings of Ufimskaya *cv*, treated with different concentrations of exogenous auxin: (**a**) main root and shoot length; (**b**) number and total root length. The bars indicate the mean values of 20 plants ± SEM. Different letters indicate a significant difference between the means at the level of *p* < 0.05.

Following the treatment of Ufimskaya *cv* bean seeds with a gradient of exogenous auxin concentrations, we observed a significant growth-stimulating effect at a concentration of 0.001 mg L^−1^ (Figure 1). Particularly, week-old seedlings pretreated with this concentration exhibited a remarkable increase in growth: shoot length, main root length (Figure 1a), and total root length (Figure 1b) being 27%, 33%, and 36% higher, respectively, compared to the control group. It is noteworthy that in the variant with an increase in concentration by an order of magnitude (0.01 mg L^−1^), the inhibition of shoot length (Figure 1a) and total root length (Figure 1b) by 27% and 22% was noted compared to the control group, respectively. Seed treatment with auxin solutions diluted by two orders of magnitude and concentrated by two or more orders of magnitude compared to the optimal concentration (0.001 mg L^−1^) led to the inhibition of the growth and development of seedling organs (Figure 1).

**Figure 2:**
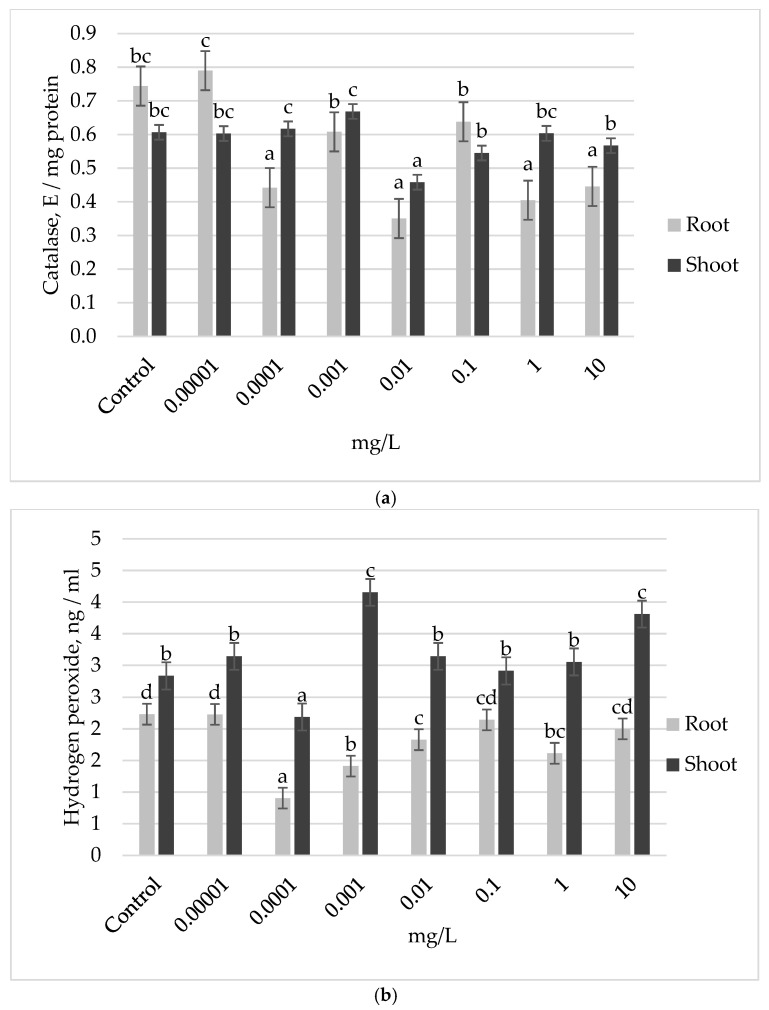

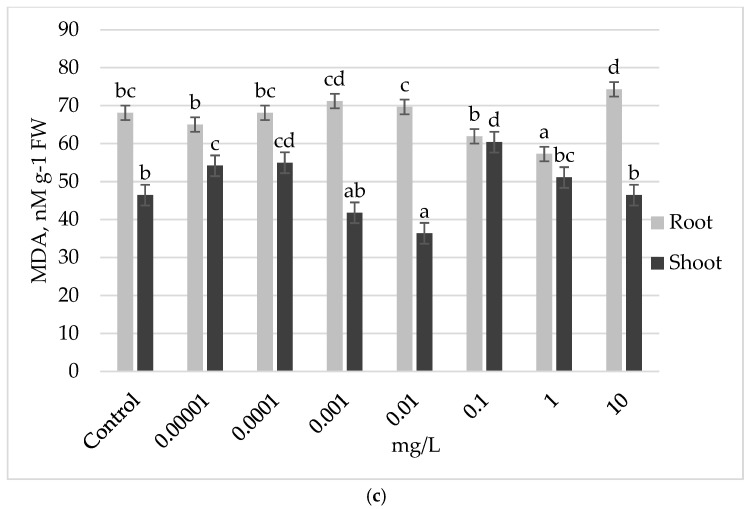
Biochemical indices of the state of antioxidant system in the roots and shoots of 7-day-old seedlings of the Ufimskaya *cv* in response to pretreatment with different doses of auxin: (**a**) catalase (CAT) activity; (**b**) hydrogen peroxide (H_2_O_2_) content; (**c**) malondialdehyde (MDA) content. The bars indicate the mean values of three repetitions ± SEM (n = 3). Different letters indicate a significant difference between the means at the level of *p* < 0.05.

Further, the state of the antioxidant system was analyzed in the roots and shoots of the Ufimskaya *cv* pretreated with different concentrations of auxin. The results showed that in response to exogenous auxin treatment, the plants exhibited a decrease in CAT activity in the roots (Figure 2a). Notably, at the optimal growth concentration of 0.001 mg L^−1^ auxin, this decrease was relatively modest (18% lower than the control). In contrast, higher auxin concentrations (10 and 100 mg L^−1^) resulted in a more significant reduction, with CAT activity declining by 28% to 53% compared to the control (Figure 2a). In the shoots, the growth-stimulating auxin concentration (0.001 mg L^−1^) showed CAT activity levels comparable to those of the control and 46% higher than those observed at the inhibitory concentration of auxin (0.01 mg L^−1^). However, this treatment also resulted in a 46% greater accumulation of H_2_O_2_ compared to the control (Figure 2b). Notably, the MDA level in this variant was close to the control level (Figure 2c). These findings suggest that obviously, at this growth stage, the shoots of week-old plants are the primary site of redox reactions of actively growing plants, pretreated with the optimal dose of exogenous auxin, compared to the non-optimal dose, and the accumulation of H_2_O_2_ in the shoots, despite the intensive work of CAT, appears to be an essential factor supporting the accelerated development of the seedlings.

Correlation analysis between morphometric and biochemical parameters of Ufimskaya cv revealed several key findings (Table 1). Shoot length correlated to a greater extent with the total root length (r = 0.94) than with the main root length (r = 0.76). Additionally, a high correlation was found between the main root length and the total root length (r = 0.76). However, growth parameters exhibited weak or no correlations with MDA and H_2_O_2_ content in roots and shoots. In contrast, a positive correlation was between shoot length and total root length with CAT activity in shoots (r = 0.67 and 0.51). There was also a negative correlation between these growth parameters and CAT activity in roots (r = −0.61 and −0.67) (Table 1).

### 2.2. Effect of Treating Elsa Cultivar Seeds with a Gradient of Exogenous Auxin Concentrations on Growth Parameters, Antioxidant System Indicators, and Correlation Relationships Between Them

**Figure 3:**
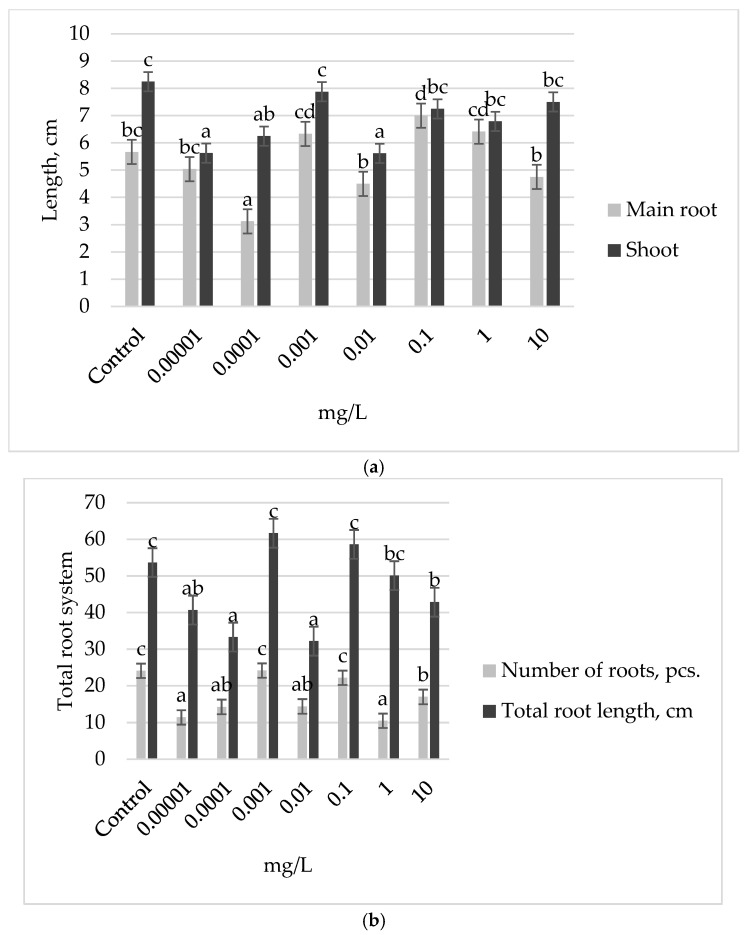
Growth parameters of 7-day-old bean seedlings of Elsa *cv*, treated with different concentrations of auxin: (**a**) main root and shoot length; (**b**) number and total root length. The bars indicate the mean values of 20 plants ± SEM. Different letters indicate a significant difference between the means at the level of *p* < 0.05.

Exogenous auxin treatment of Elsa *cv* seeds revealed that only a single stimulating concentration of the phytohormone, specifically 0.1 mg L^−1^, resulted in a 23% increase in the length of the main root compared to the control level (Figure 3). Concentrations of 0.0001 mg L^−1^ auxin were found to inhibit the growth of the main root. In contrast, treatments with concentrations of 0.001 and 10 mg L^−1^ maintained shoot length at the control level, while other concentrations resulted in a decrease in shoot growth relative to the control without inoculation. Additionally, total root length remained at the control level when treated with auxin concentrations of 0.001, 0.1, and 1 mg L^−1^; however, all other concentrations hindered the development of the root system (Figure 3b).

**Figure 4:**
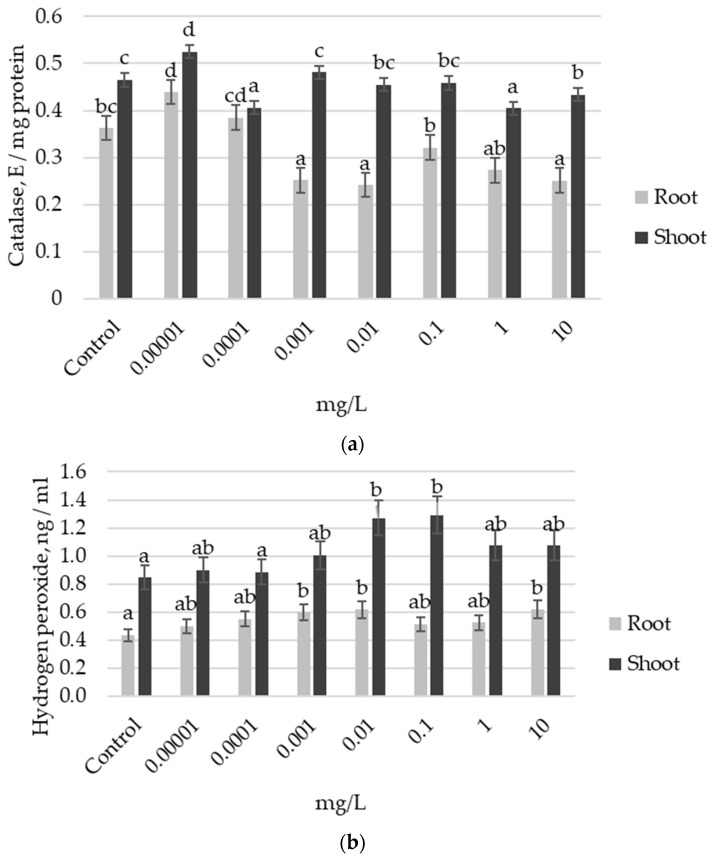

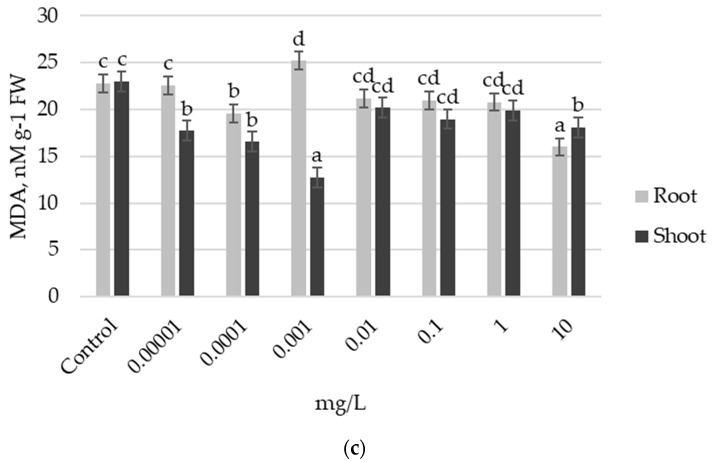
Biochemical indices of the antioxidant system in the roots and shoots of 7-day-old seedlings of the Elsa *cv* in response to pretreatment with different doses of auxin: (**a**) catalase activity, (**b**) hydrogen peroxide activity, (**c**) MDA content. Different letters indicate a significant difference between the means at the level of *p* < 0.05.

It was found that in the roots of Elsa *cv*, CAT activity was reduced in variants treated with exogenous auxin at concentrations 0.001, 0.01, and 10 mg L^−1^ (Figure 4a). Concurrently, H_2_O_2_ content in these roots increased by 40% compared to the control. In the shoots, CAT activity also decreased with treatments of 0.0001, 1, and 10 mg L^−1^; however, H_2_O_2_ content increased in the variants treated with 0.01 mg L^−1^ and 0.1 mg L^−1^ (Figure 4b). Notably, the highest MDA content level in the roots in the variant of treatment with 0.001 mg L^−1^ IAA (Figure 4c) coincided with the variant of the highest total root length (Figure 3b).

The correlation coefficients between the growth parameters of the Elsa cultivar were high and closely to Ufimsksya *cv*, when compare shoot length with total roots length (r = 0.87). But between shoot length and main root length correlation of Elsa cultivar (r = 0.46) was less close than of Ufimskaya *cv* and it differed from Ufimskaya *cv* in comparison of shoot length and number of roots (r = 0.60, it was absent at Ufimskaya *cv*). The shoot length positively correlated with CAT activity in the shoots (r = 0.58), but did not correlate with H_2_O_2_ content, and the total root length positively correlated with the catalase activity in the shoots (r = 0.60). It was almost the same at Ufimsakaya *cv*. The difference from the Ufimskaya *cv* consisted in a middle positive correlation coefficient between the MDA content in the roots and the total length of the roots (r = 0.60), while the same connection wasn’t at Ufimskaya *cv* (Table 2).

### 2.3. Effect of Treating Zolotistaya Cultivar Seeds with a Gradient of Auxin Concentrations on Growth Parameters, Antioxidant System Indicators and Correlation Relationships Between Them

**Figure 5:**
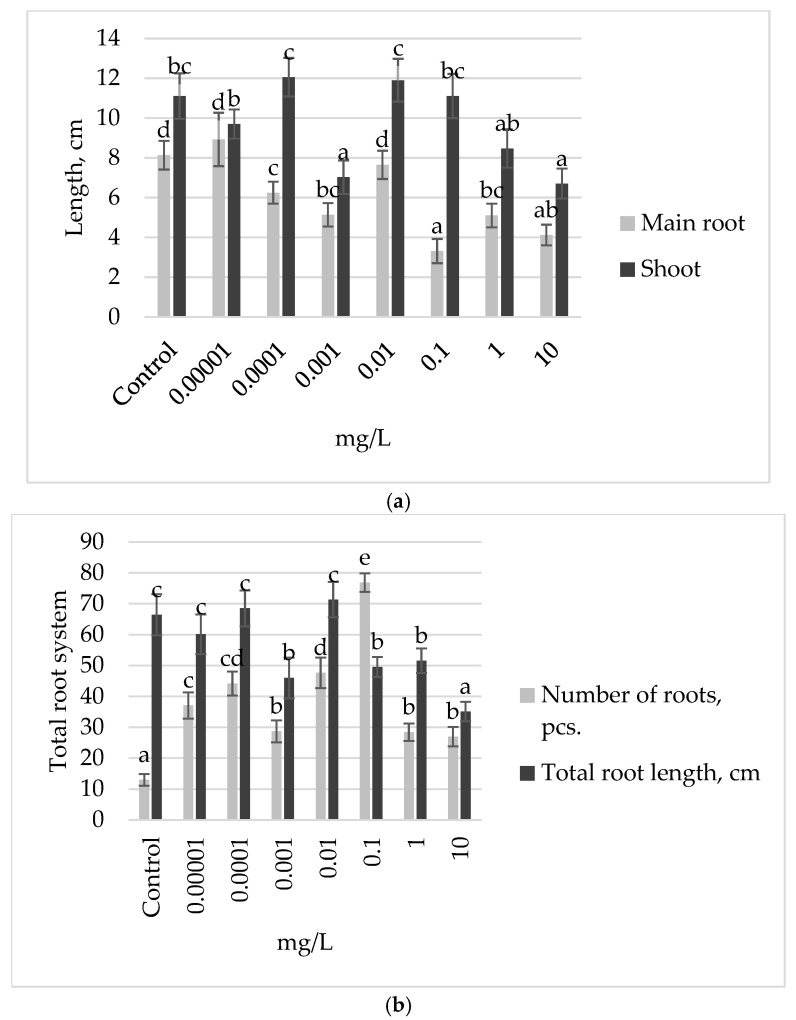
Growth parameters of 7-day-old bean seedlings of Zolotistaya *cv*, treated with different concentrations of IAA: (**a**) main root and shoot length; (**b**) number and total length of root system. The bars indicate the mean values of 20 plants ± SEM. Different letters indicate a significant difference between the means at the level of *p* < 0.05.

Seed treatment with exogenous IAA in a concentration of 0.001 mg L^−1^, which stimulated the growth of the Ufimskaya *cv*, had a negative effect on the growth of the length of both the main root and the shoot in the Zolotistaya *cv* seedlings. Other doses of 0.0001, 0.1, 1 and 10 mg L^−1^ of IAA also had an inhibitory effect on the growth of the main root, as well as the doses 1 and 10 mg L^−1^ of IAA had an inhibitory effect on shoots growth (Figure 5a). It is interesting to note that all doses of the phytohormone contributed to an increase in the formation of adventitious roots, their number increased by 2–4 times compared to the control, but their length did not exceed the control level (Figure 5b).

**Figure 6:**
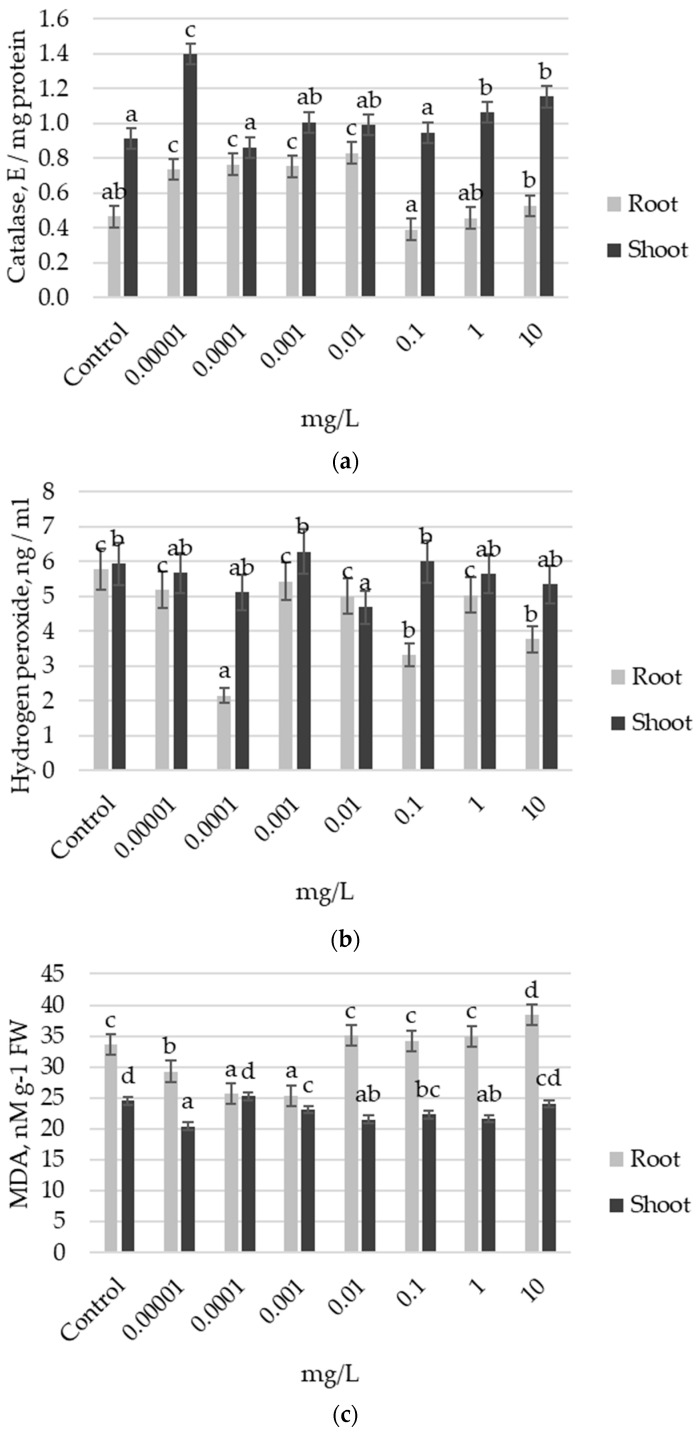
Biochemical indices of the antioxidant system in the roots and shoots of 7-day-old seedlings of the Zolotistaya *cv* in response to pretreatment with different doses of IAA; (**a**) catalase activity; (**b**) hydrogen peroxide content; (**c**) MDA content. Different letters indicate a significant difference between the means at the level of *p* < 0.05.

In the variants of phytohormone treatments with concentrations of 0.00001–0.01 mg L^−1^, the CAT activity in the roots was 1.6 times higher than the control, and in the cases of treatments with the minimum and maximum concentrations, the enzyme activity was 1.6 times higher than the control in the shoots (Figure 6a). The H_2_O_2_ content in the roots of plants pretreated with concentrations of 0.0001, 0.1 and 10 mg L^−1^ was 2–3 times lower than in the control, in the shoots a reduced level in relation to the control by 16 and 21% was noted in the variants of treatment with 0.0001 and 0.01 mg L^−1^ (Figure 6b). In the roots, a 13–24% reduction in the MDA content relative to the control was observed with heteroauxin treatments at concentrations of 0.00001–0.001 mg L^−1^; in the shoots, a 17% reduction in the MDA content was observed in the 0.00001 mg L^−1^ treatment variant (Figure 6c).

The high correlation coefficient between the shoot length and the total root length in the Zolotistaya *cv*, 0.87, completely coincided with that in the Elsa *cv*. The correlation coefficient between the shoot length and the length of the main root in the Zolotistaya *cv* was 0.41. The correlation coefficients between the length of the main root and the total root length of the Zolotistaya *cv* was 0.74. The shoot length did not correlate with biochemical parameters, and the main root length was positively connected with the content of H_2_O_2_ in the roots (r = 0.47) and CAT activity (r = 0.50) (Table 3).

A comparative analysis of Ufimskaya, Zolotistaya, and Elsa *cvs* response to exogenous auxin treatment showed that they had different stimulating concentrations: 0.001, 0.01, and 0.1 mg L^−1^, respectively. The Ufimskaya *cv* in response to exogenous auxin treatment was distinguished by a balanced growth of the root system and shoot, and its growth parameters negatively correlated with CAT activity in roots and positively correlated with CAT activity in shoots. The Ufimskaya and Zolotistaya *cvs* had in common the absence of a correlation between the number of roots and the shoot length, as well as between MDA content in roots and the growth parameters. These parameters of Elsa *cv* were connected by positive correlation coefficients. Only in the Zolotistaya *cv* the CAT activity in the roots positively correlated with the length of the main root and the total root length. At the same time, the H_2_O_2_ content in the roots positively correlated with the length of the main root and negatively with the number of roots. The individual correlations between the growth and biochemical parameters of plants in response to exogenous auxin treatment for each cultivar indicate different mechanisms of interaction between the antioxidant and phytohormonal systems for each cultivar.

Thus, the obtained results on the varietal specificity of the antioxidant system’s participation in seed treatment with a phytohormone IAA are useful for creating a model of optimal dose-dependent interaction of endophytic bacteria with bean plants to improve the efficiency of using bacterial preparations in plant growing.

## 3. Discussion

Biologics on the base of plant growth-promoting bacteria are an alternative to face the sustainability problem in agriculture, but lack of reproducible results from their application is a bottleneck for their use [10]. Auxin production is recognized as a main mechanism for plant growth promotion by beneficial bacteria; however, auxin role as a positive or deleterious factor to plant growth is dependent on concentration. The final effect of auxins depends on a fine balance of its content in plant tissues, including both bacterial and plant phytohormone production as part of complex physiological processes of plant–microbe interactions [16]. Since auxin as a signaling molecule can interfere with the plant immune system such as pathogen-associated molecular patterns, it is involved both in the mediation of the plant defensive response and in symbiosis formation [14,19,33]. The intensity of these response reactions can be monitored by the activity of the antioxidant system [34]. In this study, the interest was in comparing the growth parameters and the response of the antioxidant system of three bean varieties that differ in their adaptive properties and in their interaction with auxin-producing endophytic strains *B. subtilis*.

It was revealed earlier that the Ufimskaya *cv* showed the high stability of seed productivity in contrasting hydrothermal field conditions (adaptability coefficient 1.26); the Elsa *cv* was more dependent on favorable conditions and formed a high yield only with sufficient moisture supply (adaptability coefficient 0.72); the Zolotistaya *cv* occupied an intermediate position between the Ufimskaya and Elsa *cvs* in its adaptive properties (adaptability coefficient 0.84) [8]. It is interesting, that for the cultivars Ufimskaya—Zolotistaya—Elsa, the stimulating concentrations of exogenous auxin increased in the order of a decreasing adaptability coefficient: 0.001–0.01–0.1 mL L^−1^. It is important to note that the concentration that stimulated three growth parameters of the Ufimskaya cultivar (0.001 mL L^−1^) had an inhibitory effect on the same parameters of the Zolotistaya cultivar and a neutral effect on those of the Elsa cultivar. Therefore, it is necessary to select an individual concentration of exogenous auxin and therefore the doses of auxin-producing bacteria for each cultivar.

In this study, a nonlinear dependence of plant growth parameters on the concentration of auxin, with which bean seeds were treated, was revealed; one or two pessimums were noted in the middle of the concentration curve. A nonlinear relationship between root growth and the dose of auxin-producing bacteria, simulating the “dose effect” of exogenous auxin application, was found also in a study with cucumber plants [16]. The absence of a direct relationship between the level of phytohormone synthesis and the ability of bacteria to positively influence plant growth, as well as the species specificity of the plant response to bacterial treatment with auxin-producing bacteria, was shown in experiments with forage grasses [35]. Inhibition of root growth by 50% relative to the control at all concentrations of IAA above 1 μmoL was shown in studies with duckweed Lemma minor grown on a medium containing IAA in the range of 0–50 μmoL [36]. For cotton plants, the optimal dose of exogenous auxin was 20 mg L^−1^ (with immersion for 24 h and three-fold washing) [37]. Hargal plants (*Solenostemma Argel* (Del.) Hayne) responded positively to exogenous auxin treatment (seed immersion for 12 h) at a dose of 0.285 mM, significantly increasing water absorption, shoot length, root length, and root fresh weight after 24 h under saline conditions [38]. Thus, the absence of stimulating concentrations of exogenous auxin for some growth parameters of the Elsa and Zolotistaya cultivars, found in this study, as well as strictly defined stimulating doses of exogenous auxin are confirmed by the literature data.

Each of the studied bean cultivars was characterized by an individual growth strategy and responded differently to pre-sowing seed treatment with a gradient of IAA concentrations. The growth parameters of Ufimskaya *cv* were closely correlated with each other. This made it possible to trace closer correlations between growth parameters and biochemical indicators in seedlings. Shoot length, main root length, and total root length negatively correlated with CAT activity in roots; the first and the third parameters positively correlated with CAT activity in shoots. It is important to note that CAT activity in roots and shoots of week-old seedlings were exactly the same way negatively (in roots) and positively (in shoots) correlated with final seed productivity, when the Ufimskaya *cv* was inoculated with different auxin-producing bacteria. The decrease in CAT activity in roots was chosen as a predictor of effective symbiosis [29]. In the same way, as in the experiment with bacterial inoculation, a decrease in catalase activity in roots and its increased level in shoots correlated with growth indicators (shoot length and total root length) when seeds were treated with a gradient of IAA concentrations. However, MDA and hydrogen peroxide content in plants treated with exogenous auxin did not correlate with plants’ growth performance, whereas these very parameters correlated with the final seed productivity of plants that were treated with endophytic bacteria [29]. This allows us to make a preliminary conclusion that predictors of an effective symbiosis of Ufimskaya cultivar, H_2_O_2_, and MDA content, can be directly related to the participation of bacteria, independent of their auxin production, while the level of CAT activity may be the result of the influence of exogenous auxin action.

The Ufimskaya and Zolotistaya *cvs* had in common the absence of a correlation between the number of adventitious roots and the shoot length, unlike the Elsa *cv*, which had middle coefficients between these parameters. This could be due to different growth rates of the cultivars, which was noticed earlier [28]. The Ufimskaya and Zolotistaya *cvs* developed faster, and during the period of measuring the parameters, the plants were lengthening the already laid adventitious roots, while in the Elza *cv*, it was exactly during this period that the process of laying the peripheral root system occurred. The intensity of the process of formation of adventitious and lateral roots in the seedlings of the Elza *cv* can also be indirectly indicated by a higher correlation coefficient between the MDA content in the roots and the number of roots, as well as with other growth parameters.

There is an idea that the MDA content may reflect not only the presence of stress and membrane damage in cells but also serve as a signaling factor for the processes that accompany active growth and development of tissues [39]. The formation of lateral and adventitious roots is precisely the process in which natural membrane ruptures occur, and an increase in MDA content may be associated with these processes.

The CAT activity in the roots positively correlated with the length of the main root and the total root length only in the Zolotistaya *cv*. At the same time, this cultivar showed a positive average correlation between the content of H_2_O_2_ in the roots and the length of the main root, but an average negative correlation between the content of H_2_O_2_ and the number of adventitious roots. These features, which distinguish the Zolotistaya *cv*, can be associated with the formation of a large number of roots during the measurement period, which was characteristic only for it. The number of roots in this cultivar increased 3−7 times in response to auxin treatment, depending on the phytohormone concentration.

In this study, the stimulating dose of exogenous IAA for the growth parameters of the Ufimskaya cultivar was determined. It can serve as a reference point for determining the dose of IAA-producing strains that have a beneficial effect on the growth of plants of this cultivar. As for the Elsa and Zolotistaya *cvs*, the novel data on the sensitivity of these cultivars to exogenous auxin, revealed in this study, indicate that there is a high probability that inoculation with IAA-producing strains will not be successful. And it is necessary to seek strains with other traits for mutualistic endophytic symbiosis. The revealed absence of MDA and H_2_O_2_ content correlation with growth parameters under exogenous IAA treatment and the presence of one inoculated with endophytes plants provides a theoretical basis for exploring the mechanisms of endophytic symbiosis.

## 4. Materials and Methods

### 4.1. Plant Material and Exogenous Auxin

The studies were carried out on common bean plants (*Phaseolus vulgaris* L., Ufimskaya, Elsa, Zolotistaya cultivars). The seeds were of 2023 reproduction. Synthetic auxin indole-3-acetic acid (IAA) (PanReac, BioloT) was used as an exogenous auxin for seed treatment.

### 4.2. Preparation of the IAA Concentration Gradient

Initially, 2 mg of dry IAA was weighed and dissolved in 100 µL of 96% ethanol. The volume of this solution was then gradually increased to 20 mL by adding distilled water, resulting in a final IAA concentration of 100 mg L^−1^. Subsequently, this solution was subjected to a series of 10-fold dilutions, producing concentrations ranging from 10 mg L^−1^ down to 0.00001 mg L^−1^.

### 4.3. Seed Treatment

The seeds were sterilized using brilliant disinfectant (0.9% alkyldime-thylbenzylammonium chloride and 0.8% glutaraldehyde) for 10 min, followed by repeated washing with distilled water. For each experimental variant, 20 bean seeds were soaked in 4 mL of IAA solutions in a range of concentrations (0, 10, 1, 0.1, 0.01, 0.001, 0.0001, 0.00001 mg L^−1^) in Petri dishes for 3 h. The treated seeds were then placed on moistened filters in plastic containers (size 15 × 20 × 15 cm) with lids. In the control group, the seeds were soaked in sterile water. All seeds were germinated in the dark at 22–24 °C.

### 4.4. Determination of Seedlings Growth

Growth parameters were evaluated on 20 plants that were 7 days old. The assessed growth parameters included the length of the main root and shoot, as well as the number and length of all roots.

### 4.5. Determination of Malondialdehyde (MDA) Content

The content of MDA was assessed by evaluating its reaction with thiobarbituric acid [40]. A frozen sample (0.25 g) of plant tissue was homogenized with 3 v of ice-cold 10% trichloroacetic acid (TCA) and then centrifuged at 10,000× *g* for 15 min. The assay mixture was prepared by combining a 1 mL aliquot of the supernatant with 1 mL of 0.5% (m/v) 2-thiobarbituric acid in 20% (m:v) TCA. This mixture was heated to 95 °C for 1 h and then rapidly cooled in an ice bath. The absorbance of the supernatant was measured at 532 nm and 600 nm using a SmartSpecPlus spectrophometer (Bio-Rad, Hercules, CA, USA). MDA content was calculated using an absorbance coefficient of 155 mmoL^−1^ cm^−1^.

### 4.6. Determination of Hydrogen Peroxide (H_2_O_2_) Content

The content of H_2_O_2_ in plant extracts was measured following the method [41]. Plant tissues were homogenized in a 0.01 moL potassium phosphate buffer (pH 6.2), using a tissue-to-buffer ratio of 1:5 (m:v). The homogenates were extracted for 10–15 min at 4 °C, after which they were centrifuged at 4 °C for 10 min at 10,000× *g*. A volume of 50 μL of the supernatant was then taken to initiate the reaction in a total mixture volume of 150 μL, which contained 25 mM FeSO_4_ and 25 mM (NH_4_)_2_SO_4_ dissolved in 2.5 mM H_2_SO_4_. Subsequently, 1 mL of this solution was added to 100 mL of a 125 μM xylenol orange solution (Merk, Darmstadt, Germany) along with 100 mM sorbitol. The H_2_O_2_ content was assessed using xylenol orange dye, as hydroperoxides react with ferrous ions in an acidic solution to form a ferric product-xylenol orange complex. The reaction mixture was incubated in the dark at room temperature for 1 h, and the absorbance was measured at 570 nm using an EnSpire 2300 spectrophotometer (Perkin Elmer, Waltham, MA, USA). The H_2_O_2_ content was calculated using a standard curve derived from the absorbance values of H_2_O_2_ standards.

### 4.7. Determination of Catalase (CAT) Activity

The activity of CAT (EC 1.11.1.6) was assessed by measuring the breakdown of H_2_O_2_, as described [42]. Plant tissues were homogenized in a 0.05 M potassium phosphate buffer (pH 7.8) at a ratio of 1:5 (m:v). The resulting mixture was then centrifuged at 4 °C for 10 min at 13,000× *g*. The reaction was initiated by adding 0.15 mL of a 0.03% H_2_O_2_ solution to 0.02 mL of the enzyme extract in wells of a flat-bottomed immunoassay plate (Costar, Washington, DC, USA). After 1 min, the reaction was halted by the addition of 0.075 µL of 4% (NH_4_)6Mo_7_O_24_ × 4H_2_O. The activity of CAT was measured using a Benchmark microplate reader (Bio-Rad, Hercules, CA, USA) by monitoring the change in absorbance at 405 nm, which indicates the extinction of H_2_O_2_ complexes with molybdenum ions. Total protein content was quantified according to the Bradford method [43], in wells of a flat-bottomed plate for immunoassay (Costar, Washington, DC, USA), with chymotrypsin as the standard by spectrophotometer- fluorimeter Feyond-A400 (Allsheng, Hangzhou, China) at 595 nm.

### 4.8. Statistical Analysis

The experiments were conducted in three biological and three analytical replicates. For the analysis of variance (ANOVA) between the treatment groups, the statistical analysis was performed using the Microsoft Office Excel 2010 package. The data presented are mean values with standard errors (±SE). The calculation of Pearson’s correlation coefficients and the construction of correlation matrices were carried out using the Data Analysis ToolPak in Excel 2016.

## 5. Conclusions

The findings showed dose-dependent and cultivar-specific responses of 7-day-old bean seedlings to exogenous IAA. Ufimskaya cv exhibited significant increases in shoot, main root, and total root lengths at 0.001 mg L^−1^ IAA, while higher and lower concentrations inhibited growth. The reduced catalase (CAT) activity in roots and the elevated CAT activity in shoots correlated with shoot length and total root length of Ufimskaya cv. Importantly, the growth parameters exhibited weak or no correlations with malondialdehyde (MDA) and H_2_O_2_ content in roots and shoots, which is a peculiarity of the Ufimskaya cv response to exogenic IAA in contrast to the shown earlier response to inoculation with endophytes. The growth of only the main root of Elsa cv. peaked at 0.1 mg L^−1^ IAA, and there were neutral or inhibitory effects with other concentrations. The positive correlation between CAT activity in shoots and the main root length and total root length as well as positive correlation between MDA content in roots and the total root length of Elsa cultivar were revealed. The shoot length and total root length of Zolotistaya cv were neutral or negatively responded to all concentration IAA, but the number of roots increased by 2–4 times. For Zolotistaya cv, positive correlations were observed between CAT activity in roots and the length of the main root and the total length of the roots.

This study provides valuable insights into the complex interplay between exogenous IAA application and antioxidant system responses in three bean cultivars: Ufimskaya, Elsa, and Zolotistaya. Here, the treatment with a gradient of IAA concentrations served as a mechanistic model of the interaction of plants with endophytic bacteria, as if this influence were limited exclusively to the production of bacterial auxin. At the same time, we assumed that the hormone can act not only as a growth regulator but also as a signaling factor mediating symbiotic relationships. The identified different levels of exogenous IAA for each cultivar can be useful for further precision design of biopreparations, most suitable for a specific cultivar, and this is very important from a practical point of view. The curious thing is that the predictors of effective endophytic symbiosis, identified in our previous work for Ufimskaya cultivar, such as reduced MDA levels and increased hydrogen peroxide levels in roots, were not associated (not correlated) with seedlings growth when they were pretreated with pure auxin without bacteria. This provides new ways of speculation about the mechanisms of interaction between plants and endophytes. The differences in antioxidant system responses reflect individual pathways of development and shaping of cultivars adaptive strategies, that endophytic bacteria can intervene in. The divergent responses observed among the cultivars underscore the importance of understanding the intricate balance between phytohormone signaling and the antioxidant defense mechanisms in plant–microbe interactions. These findings highlight the need to tailor auxin-producing bacteria inoculum levels to specific cultivars for consistent growth promotion. Further research into the molecular and physiological mechanisms behind these cultivar-dependent responses could enhance plant–microbe interactions and improve agricultural sustainability.

## Figures and Tables

**Table 1:** Correlation coefficients between growth indicators and biochemical indicators of the antioxidant system in 7-day-old bean seedlings of Ufimskaya cultivar.

	Main Root	Shoot	Number of Roots	Total Root Length	CAT Root	CAT Shoot	H_2_O_2_ Root	H_2_O_2_ Shoot	MDA Root	MDA Shoot
Main root	1.00									
Shoot	0.76	1.00								
Number of roots	0.35	0.25	1.00							
Total root length	0.76	0.94	0.37	1.00						
CAT root	−0.51	−0.61	−0.23	−0.67	1.00					
CAT shoot	0.22	0.67	0.01	0.51	−0.09	1.00				
H_2_O_2_ root	0.02	−0.15	−0.06	−0.08	0.06	−0.36	1.00			
H_2_O_2_ shoot	−0.18	0.09	−0.06	0.14	−0.07	0.19	0.13	1.00		
MDA root	−0.38	−0.13	−0.17	−0.31	0.06	0.01	−0.15	0.46	1.00	
MDA shoot	−0.40	−0.22	−0.09	−0.26	0.05	0.28	−0.13	−0.29	−0.29	1.00

**Table 2:** Correlation coefficients between growth parameters and biochemical indicators of the antioxidant system in bean seedlings of Elsa *cv*.

	Main Root	Shoot	Number of Roots	Total Root Length	CAT Root	CAT Shoot	H_2_O_2_ Root	H_2_O_2_ Shoot	MDA Root	MDA Shoot
Main root	1.00									
Shoot	0.46	1.00								
Number of roots	0.44	0.60	1.00							
Total root length	0.79	0.87	0.62	1.00						
CAT root	−0.14	0.21	−0.10	0.17	1.00					
CAT shoot	0.30	0.58	0.23	0.60	0.57	1.00				
H_2_O_2_ root	−0.32	−0.34	−0.18	−0.40	−0.72	−0.33	1.00			
H_2_O_2_ shoot	0.33	−0.19	0.00	−0.02	−0.59	−0.16	0.47	1.00		
MDA root	0.45	0.38	0.38	0.60	0.33	0.64	−0.40	−0.23	1.00	
MDA shoot	0.09	0.17	−0.06	0.01	0.20	0.07	−0.56	0.06	−0.12	1.00

**Table 3:** Correlation coefficients between growth parameters and biochemical indicators of the antioxidant system in bean seedlings of Zolotistaya cultivar.

	Main Root	Shoot	Number of Roots	Total Root Length	CAT Root	CAT Shoot	H_2_O_2_ Root	H_2_O_2_ Shoot	MDA Root	MDA Shoot
Main root	1.00									
Shoot	0.41	1.00								
Number of roots	−0.39	0.44	1.00							
Total root length	0.74	0.87	0.04	1.00						
CAT root	0.50	0.15	−0.04	0.44	1.00					
CAT shoot	0.29	−0.44	−0.15	−0.29	0.14	1.00				
H_2_O_2_ root	0.47	−0.28	−0.56	0.05	0.01	0.33	1.00			
H_2_O_2_ shoot	−0.26	−0.42	−0.13	−0.45	−0.46	0.05	0.33	1.00		
MDA root	−0.24	−0.11	0.01	−0.29	−0.57	0.11	0.12	−0.26	1.00	
MDA shoot	−0.21	0.06	−0.28	−0.01	−0.11	−0.65	−0.44	0.00	−0.16	1.00

## Data Availability

The original contributions presented in this study are included in the article. Further inquiries can be directed to the corresponding author.
